# Significant correlation of angiotensin converting enzyme and glycoprotein IIIa genes polymorphisms with unexplained recurrent pregnancy loss in north of Iran

**Published:** 2016-05

**Authors:** Shokoufeh Fazelnia, Touraj Farazmandfar,, Seyed Mohammad Bagher Hashemi-Soteh

**Affiliations:** 1 *Department of Biology, Damghan Branch, Islamic Azad University, Damghan, Iran.*; 2 *Medical Cellular and Molecular Research Center, Golestan University of Medical Sciences, Gorgan, Iran.*; 3 *Immunogenetic Research center, Molecular and cell Biology Research Centre, Mazandaran University of Medical Sciences, Sari, Iran.*

**Keywords:** *Angiotensin converting enzyme*, *Platelet glycoprotein IIIa*, *Recurrent abortion*

## Abstract

**Background:**

Spontaneous abortion is considered as the most complex problem during pregnancy. Thrombophilia is resumed as a cause of recurrent pregnancy loss (RPL). Glycoprotein IIIa (GPIIIa) gene is involved in thrombosis and abortion. Angiotensin converting enzyme (ACE) converts angiotensin I to angiotensin II and is involved in thrombosis. The most common polymorphism in this gene is the insertion/deletion (I/D).

**Objective:**

In this study, we analyzed the association between *ACE* I/D and *GPIIIa* c.98C >T polymorphisms in women with unexplained RPL from the north of Iran.

**Materials and Methods:**

Sample population consisted of 100 women with unexplained RPL and 100 controls. The *ACE* I/D and *GPIIIa* c.98C>T polymorphisms were genotyped by TETRA-ARMS PCR. The association between genotypes frequency and RPL were analyzed using χ^2^ and exact fisher tests. Associated risk with double genotype combinations was also investigated by binary logistic regression.

**Results:**

There was significant association between *ACE* DD genotype and RPL (OR=2.04; 95% CI=0.94-4.44; p=0.036). *ACE* D Allele was also significantly associated with the RPL (OR=1.59; 95% CI=1.05-2.41; p=0.013). No significant association was observed between *GPIIIa* c.98C>T polymorphism and RPL.

**Conclusion:**

*ACE* I/D polymorphism may probably be a prognostic factor in female family members of women with the history of recurrent abortion.

## Introduction

Recurrent pregnancy loss (RPL) appears a signiﬁcant clinical problem affecting approximately 2% of women (1). RPL pathophysiology is poorly understood. Pregnancy loss may be caused by different reasons such as genetic factors, immune defect, infection and anatomical problem (2). However, even after more accurate investigations, as many as 50% of all cases remain unexplained (3). Thrombophilia has been presumed as a cause of RPL (4, 5). Many recent studies have examined the mutations incidence and variants in speciﬁc thrombophilic genes on women with unexplained pregnancy loss (4-10). Angiotensin converting enzyme (ACE) is a key component in rennin-angiotensin system which converts angiotensin I to angiotensin II, a potent vasopressor. Many studies have been indicated that ACE affects hemostasis through different mechanisms, including platelet aggregation, blood clotting and ﬁbrinolysis (11-14). 

The human *ACE* gene contains variable polymorphic regions that can be used in genetic analysis. A well-known polymorphism is the insertion/deletion (I/D) of 287 base paired (bp) fragment in intron 16 which has been extensively investigated (1). Furthermore, it has been found that *ACE* D allele leads to increased expression in plasminogen activator inhibitor-1 (PAI-1) level, which can increase the angiotensin II production and enhance the thrombotic events risk (15, 16). 

Past studies have been indicated that thrombophilic defects affect pregnancy-associated thromboembolism such as preeclampsia and abortion (11). Few recent meta-analysis studies have demonstrated the role of *ACE* I/D polymorphism in increasing RPL risk (17, 18). Glycoprotein IIIa (*GPIIIa*) is one of thrombophilic genes involved in modulation of vascular thrombosis. The GPIIb/GPIIIa is an integrin complex in platelet aggregation as a ﬁbrinogen receptor (19). The polymorphism c.98C>T in *GPIIIa* gene causes an amino acid substitution (p.L33P), leads to the creation of two distinct forms of GPIIb/IIIa antigen on platelets (20). This polymorphism has been associated with stroke in young Caucasian women and risk of premature acute coronary syndromes (21). This variation also results in spiral artery thrombosis and poor placental perfusion, which can explain the correlation of this polymorphism with RPL (22, 23).

To date, few studies have established on the relationship between thrombophilic genes and RPL in Iranian population (24-26). We, therefore, determined the association between of *ACE* I/D and *GPIIIa* c.98C>T polymorphisms in women from the northern of Iran with unexplained RPL.

## Materials and methods

**Subjects**

This case-control study was done carried out during the 2013-2014 year’s in Sari, Iran. A total of 100 women with unexplained RPL aged 20-40 yrs and 100 healthy controls aged 27-44 yrs, with at least two live births and no history of abortion, infertility or endometriosis were comprised. Prior to enrollment, all patients were given an explanation of study nature, and written informed consent was obtained from all individuals. The study protocol was approved by the Clinical Research Ethics Committee in Mazandaran University of Medical Sciences, Sari, Mazandaran. RPL was defined as two or more spontaneous consecutive abortions at 5-20 wks of gestation. Miscarriage history of women with unexplained RPL was examined and cases with anatomic, chromosomal, hormonal, autoimmune or infectious causes were excluded from this study. There was no pregnancy-related problems such as hypertension, diabetes, thyroid abnormalities, etc. in none of cases. 

**Genotyping**

Genomic DNA was isolated from whole blood by a modified Nucleon BACC II method from whole blood (Tepnel Life Sciences, Manchester, UK). The *ACE* I/D polymorphism was genotyped using two primers and *GPIIIa* c.98C>T polymorphism was genotyped using a TETRA-ARMS PCR method (27, 28). The *GPIIIa* c.98C>T polymorphism primers were designed by Gene Runner software (version 3.05) (Table I). The polymerase chain reaction (PCR) was performed using standard PCR methods with 100-200 ng of DNA template and 5 picomol of each primer and PCR materials (Cinnagen, Tehran, Iran) in thermal cycler (Eppendorf, Hamburg, Germany) as reported previously (29). 

PCR conditions for *ACE* genotyping included one step initial denaturation (94^o^C for 3 min), 35 cycles (94^o^C for 45 sec, 60.5^o^C for 50 sec, and 72^o^C for 40 sec) and a final extension at 72^o^C for 5 min. PCR conditions for *GPIIIa* genotyping also included one step initial denaturation (94^o^C for 3 min), 35 cycles (94^o^C for 35 sec, 58^o^C for 40 sec, and 72^o^C for 40 sec) and a final extension at 72^o^C for 5 min. Then, PCR products were electrophoresed in a 1.5% agarose gel (Fermentas, Sankt Leon-Rot, Germany). 

**Statistical analysis**

Association between genotypes frequency and RPL were analyzed using  ^2^ and Fisher’s exact tests. The risk associated with the double genotype combinations was also investigated by binary logistic regression. Data were analyzed by SPSS software (version 17.0) and p˂0.05 were considered significant.

## Results

**Figure 1:**
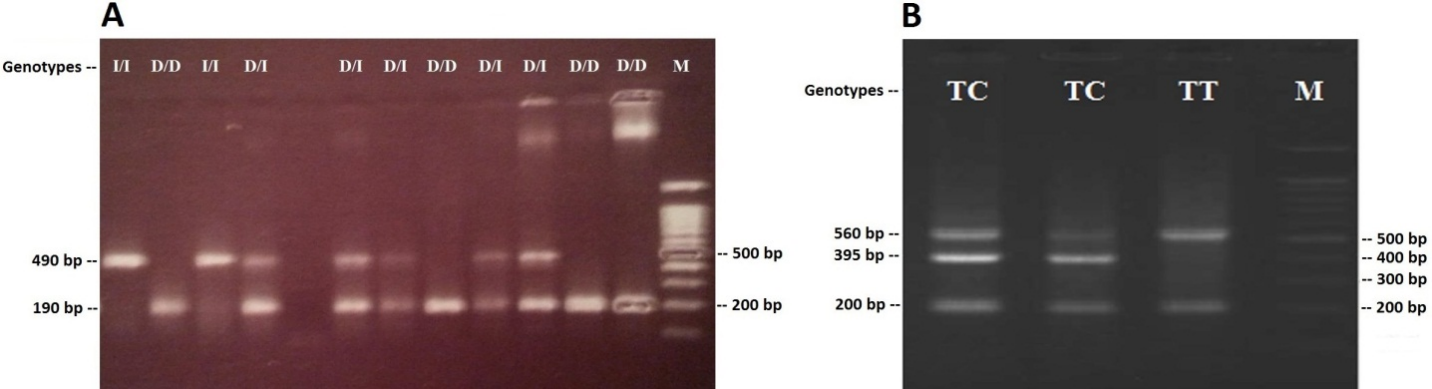
Electrophoresis pattern of PCR products for detection of polymorphisms.(A)ACE I/D polymorphism. Band of 490 bp and 190 bp are as I allele and D allele respectively. (B)GPIIIa c.98C>T polymorphism. Band of 395 bp and 200 bp are as C allele and T allele respectively. Band of 560 bp is as control

The *ACE* I/D Alleles were determined based on Product size-band; 490 bp for the I allele and 190 bp for D allele (Figure 1A). The *GPIIIa* c.98C>T Alleles were also determined by bands; 395 bp for the C allele (by primers of reverse outer and forward inner), 200 bp for T allele (by primers of forward outer and reverse inner) and 560 bp as control (by primers of outer) (Table I) (Figure 1B). The *ACE* I/D and *GPIIIa* c.98C>T genotypes in women with RPL and control group was in accordance with Hardy-Weinberg equilibrium. A few samples from each genotype were confirmed by sequencing. The Genotypic and allelic frequencies of *ACE* I/D and *GPIIIa* c.98C>T polymorphisms in women with RPL and control group were shown in tables II and III. The *ACE* II genotype was observed in 23% of cases, *ACE* ID genotype in 33% and *ACE* DD genotype in 44%. The *GPIIIa* c.98C>T wild-type genotype (TT) was observed in 84% of cases, whereas 16% were heterozygous (TC) and no CC genotype was observed. Our results showed that, there is a significant difference regarding *ACE*DD genotype between cases and control groups (OR=2.04; 95% CI=0.94-4.44; p=0.036) (Table II). 

Our results also indicated that D Allele is statistically associated with RPL (OR=1.59; 95% CI=1.05-2.41; p=0.013) (Table III). No significant difference was observed between genotypic and allelic frequencies of *GPIIIa* c.98C>T polymorphism and RPL in case and control groups. In combination analysis, there was no significant association between combination of *ACE*DD genotype and *GPIIIa* TT genotype with RPL (Table IV).

**Table I:** Primers used for genotyping

**Primer**		**Sequence (5´3´)**	**Genbank accession No.**
ACE I/D			NG_011648.1
	Forward	CTGGAGACCACTCCCATCCTTTCT	
	Reverse	GATGTGGCCATCACATTCGTCAGAT	
GPIIIa c.98C>T			NG_008332.2
	Forward outer	CCTTTCTGTACAACGGTCCT	
	Reverse outer	CAGATCTTCTGACTCAAGTCCT	
	Forward inner (C)	CTTACAGGCCCTGCGTCC	
	Reverse inner (T)	CACAGCGAGGTGAGCACA	

**Table II:** Genotype frequencies of ACE I/D and GPIIIa c.98C>T polymorphisms in women with RPL. The risk of I/I versus (I/D + D/D) and (I/I + I/D) versus D/D for RPL was evaluated in dominant and recessive models (n=100)

		**Case **	**Control **	**OR (95% CI)**	**p-value**
ACE I/D					
	II	23	31	1.00	
	DI	33	40	1.11 (0.51 - 2.40)	0.455
	DD	44	29	2.04 (0.94 - 4.44)	0.036^$^
	DI + DD	77	69	1.50 (0.76 - 2.97)	0.104
	DD	44	29	1.00	
	DI	33	40	0.54 (0.26 - 1.10)	0.097
	II	23	31	0.48 (0.22 - 1.06)	0.071
	DI+II	56	71	0.51 (0.28 - 0.93)	0.131
GPIIIa c.98C>T					
	TT	84	80	1.00	
	TC	16	20	0.76 (0.36 - 1.58)	0.469
	CC	0	0	-	-
	TC + CC	16	20	0.76 (0.36 - 1.58)	0.469

OR: odds ratio

CI: confidence interval

$ significant p-values

**Table III:** Allelic frequencies of ACE I/D and GPIIIa c.98C>T polymorphisms in women with RPL

		**Case **	**Control **	**OR (95% CI)**	**p-value**
ACE I/D					
	I	79 (39.5%)	102 (51%)	1.00	
	D	121 (60.5%)	98 (49%)	1.59 (1.05 - 2.41)	0.013^$^
GPIIIa c.98C>T					
	T	184 (92%)	180 (90%)	1.00	
	C	16 (8%)	20 (10%)	0.78 (0.38 - 1.56)	0.491

Data presented as n (%).

$ Significant p-values

OR: odds ratio

CI: confidence interval

**Table IV:** Combination analysis of ACE I/D and GPIIIa c.98C>T polymorphisms in women with RPL

		**Case **	**Control**	**OR (95% CI)**	**p-value**
ACE/ GPIIIa					
	II/TT	20	26	1.00	
	II/TC	3	5	0.78 (0.10 - 4.59)	0.389
	DI/TT	28	30	1.21 (0.51 - 2.84)	0.316
	DI/TC	5	10	0.65 (0.15 - 2.52)	0.255
	DD/TT	36	24	1.95 (0.83 - 4.57)	0.067
	DD/TC	8	5	2.08 (0.50 - 9.29)	0.136

OR: odds ratio

CI: confidence interval

## Discussion

This study was designed to determine the association between certain polymorphisms in *ACE* and *GPIIIa* genes and RPL in Mazandaran province, northern Iran. Changes in renin-angiotensin-aldosterone system are involved in pathogenesis of pregnancy complications such as preeclampsia and fetal loss (30, 31). Previous studies elucidated the role of *ACE* I/D polymorphism in susceptibility to pregnancy loss (32-36). 

ACE alters hemostasis through some mechanisms, including platelet aggregation, inﬂuence on ﬁbrinolysis. Some previous studies have reported an association between the *ACE* D allele and increased risk of thrombosis (15, 37). Data by Fatini *et al* demonstrated that the *ACE* I/D polymorphism may be an important risk factor for RPL (35). Buchholz *et al* also reported that the *ACE* DD genotype leads to increased PAI-1 concentration and thus correlated with an increased risk of RPL in Caucasians (14). However, Vettriselvi *et al* showed no signiﬁcant association between the frequencies of deletion allele and RPL (1). In this study, we found an association between *ACE* I/D polymorphism and RPL in our population. This study demonstrated that the DD genotype was more prevalent in RPL cases (44%) than in controls (29%), and D allele by itself might be a risk factor for RPL in this population. 

Concerning the *GPIIIa* c.98C>T polymorphism, it seems to increase susceptibility to premature acute coronary syndrome and risk of stroke in young Caucasian women (21). T allele frequency was low in all the past studies which is in contrast with this study (1%-17%), this may be due to small sample size in our study (36, 38, 39). Yenicesu *et al* reported that heterozygous mutations of *GPIIIa* c.98C>T (CT) were associated with RPL (38). Goodman *et al* showed that *GPIIIa* L33P can identify women at risk for RPL (7). Coulam *et al* also determined the frequency of *GPIIIa* L33P in women with RPL history (40). Our results indicated that the *GPIIIa* c.98 C>T polymorphism was not significantly correlated with RPL. These results are in agreement with the study’s results of, Ozdemir *et al*, Torabi *et al*, Pihusch *et al*, Coulam *et al*, Goodman *et al* and Hohlagschwandtner *et al* (7, 26, 39-42).

## Conclusion

The major finding of this study shows that ACE D allele may increase susceptibility to RPL. ACE I/D polymorphism could probably be investigated as one of the prognostic factors in women with RPL history among the family members.

## Note

***This article extracted from M.Sc. thesis. (Shokoufeh Fazelnia)***
